# Treatment of Lowland Frogs From the Spawn Stage with Homeopathically Prepared Thyroxin (10^-30^)

**DOI:** 10.1100/tsw.2007.220

**Published:** 2007-10-22

**Authors:** Helmut Graunke, P. Christian Endler, Waltraud Scherer-Pongratz, Heinz Spranger, Michael Frass, Harald Lothaller

**Affiliations:** ^1^ Interuniversity College for Health and Development, Graz, Castle of Seggau, Austria; ^2^ University of Graz, Austria

**Keywords:** amphibian, hormone, thyroxin, homeopathic dilution, curative effect

## Abstract

The influence of a highly diluted agitated, i.e. homeopathically prepared thyroxin solution (10^-30^, final concentration in the basin water 10^-35^ parts by weight after the first application) on metamorphosis in lowland Rana temporaria from the spawn stage on was studied. The treatment with homeopathically prepared thyroxin solution (10^-30^) starts at the frogspawn stage. It represents a tool to learn more about the previously standardized amphibian model, where the thyroxin solution was applied from the two- legged stage on only. Lowland frogs were pretreated by immersing spawn in an aqueous molecular thyroxin dilution (10^-8^ parts by weight). In later stages of development (2 to 4 legged), this has been found to speed up metamorphosis by around 15%. In accordance with the homeopathic idea of detoxication or cure, hyperstimulated animals (spawn or, in subsequence, larvae) were treated either with thyroxin that had been highly diluted and agitated in successive steps, i.e. homeopathically prepared (10^-30^), or analogously prepared blank solution (water). Development was monitored by documenting the number of animals that had entered the four-legged stage. It has been found that animals treated with the test solution metamorphosed more slowly than the control animals, i.e. the effect of the homeopathically prepared thyroxin was opposed to the usual effect of molecular thyroxin. The number of test animals that reached the 4- legged stage at defined points in time was slightly smaller in the group treated with homeopathically prepared thyroxin at some, but not at all points in time, compared to control. The results in this study sustain the previous multi researcher findings that highly diluted homeopathically prepared thyroxin is able to slow down metamorphosis of *Rana temporaria*.

### INTRODUCTION

Intoxication – detoxification experiments are an important tool in research on homeopathy[1,2]. Due to the relation between an enhanced thyroxin level during metamorphosis[3] and homeopathically prepared thyroxin, a model with amphibians seems to fit well into this category[4,5].

### Highland Animals and Thyroxin $10^{-30}$

In previous experiments[7,8,9], we used *Rana temporaria* larves from alpine highland populations, assuming that the highland animals' natural thyroxin level is comparatively high and that the increase in environmental temperature causes an artificial acceleration of metamorphosis, when the animals are brought from the highland biotopes to the laboratory. Animals were treated with homeopathically prepared dilution of thyroxin $10^{-30}$ (final concentration in the basin water $10^{-35}$) from the two-legged stage on. In five independent laboratories in Austria and the Netherlands, it was found that the dilution diminished the speed of metamorphosis (3-11%). Differences were statistically significant ($p < 0.01$ or $< 0.05$ at the measuring points in time). Frequencies of animals treated with homeopathically prepared thyroxin reached the values of control animals with a delay of about ½-1 intervals between points in time[7,8,9].

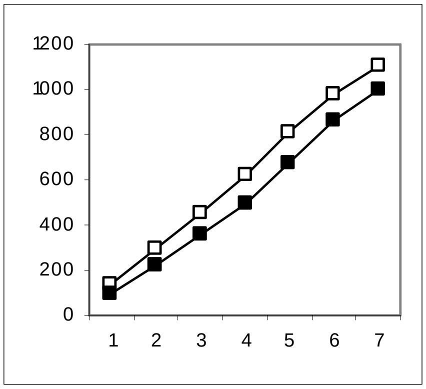

**Figure 1.** The effect on highland *Rana temporaria*, added from the two-legged stage on; pooled data from 1990 and 2000 [7-9]. Ordinate = cumulative frequency of 4- legged tadpoles (N). Abscissa = points in time. Black squares = frequencies of animals treated with homeopathically prepared thyroxin; white squares = of animals treated with analogously prepared water.

### Lowland Animals and Thyroxin $10^{-30}$

When *Rana temporaria* from lowland biotopes were used in the same experimental set-up, starting treatment at the two- legged stage, no difference was found between the test and the control group ($p > 0.05$)[6].

### Hyperstimulation

In further experiments we observed that hyperstimulation of lowland animals with molecular thyroxin (final concentration in the basin water $10^{-8}$) was able to enhance animals' responsiveness to homeopathically prepared thyroxin[6]. However, this was applicable only to treatment with homeopathically prepared thyroxin $10^{-8}$, but was not applicable to treatment of lowland animals with thyroxin $10^{-30}$. Nor did hyperstimulation enhance the observed effect of thyroxin $10^{-30}$ in highland animals[10].

In the following study, the influence of thyroxin $10^{-30}$ on metamorphosis in hyperstimulated lowland *Rana temporaria* was studied. Different to our previous studies, where treatment started at the two-legged stage, in the study presented here, animals were treated from the *spawn stage* on.

## METHODS

### Researchers and Blinding

The experiments were carried out at the laboratory of the Interuniversity College and the study planned by Graunke and Endler. Application of the homeopathically prepared thyroxin or solvent was done blind.

### Animals, Staging, Water and Further Laboratory Conditions

Four days old *Rana temporaria* spawn were taken from an Austrian lowland pool. The larvae (tadpoles) were treated and observed during seven weeks until both, the hind and forlegs, broke through the skin and the animals had thus entered the four-legged stage.

20 eggs were allotted to each of a total of 75 white plastic basins according to a random procedure. Basins contained 6 l of dwell water each. Indirect natural light was used. Room temperature was 21 + 1°C. The tadpoles were fed with blanched greens (lettuce) ad libitum. The experiment was carried out between April and June 2006.

### Preparation and Administration of Hyperstimulation and Test Solutions

Two groups of animals were exposed to the stock solution of tetra-iodo-thyronin sodium pentahydrate ($T_4$, Sigma, $10^{-4}$ parts by weight in double distilled water, diluted in the basin water down to a final concentration of $10^{-8}$) (immersion in thyroxin $10^{-8}$, hyperstimulation). This is a standard procedure in our laboratory derived from studies with *Rana temporaria* from lowland biotopes, where it speeds up metamorphosis by about 15%[6]. One group of the hyperstimulated highland amphibians was then treated with the homeopathically prepared test dilution, while the other was treated with the analogously prepared solvent:

- thyroxin $10^{-8}$ + thyroxin $10^{-30}$ (hyperstimulated test group)
- thyroxin $10^{-8}$ + water $10^{-30}$ (hyperstimulated control group)

The third group was treated with

- water $10^{-30}$ (inert control group).

For preparation of the test dilution thyroxin $10^{-30}$, the stock solution ($10^{-4}$), was diluted with pure double distilled water in 26 steps of 1:10, and agitated after each step of dilution according to a standardized protocol. Analogously prepared solvent was used as a control (water $10^{-30}$).

3 μl of molecular stock solution ($10^{-4}$) was added per animal and 300 ml of basin water of the hyperstimulated groups (final concentration of the hyperstimulation solution was $10^{-8}$ after the first application) at intervals of 2 weeks. 3 μl of probe dilution (test or control) was added per animal and 300 ml of basin water (final concentration of probe dilution was $10^{-35}$ after the first application) at intervals of 48 hours.

### Comparison and Evaluation of Data

After reaching the four-legged stage the cumulative frequency of animals treated with test or control was aggregated for each day. Treatment was started on April 29th. On June 10th the first animals in a four-legged stage were observed. According to the reasons described in study[14], chi-square tests were used to compare groups at the points in time. Further statistical methods used in connection with the amphibian model have been described in[6,9].

### RESULTS

500 animals were treated with thyroxin $10^{-8}$ plus thyroxin $10^{-30}$ (hyperstimulated test group), 500 animals with thyroxin $10^{-8}$ plus water $10^{-30}$ (hyperstimulated control group) and 500 animals with water $10^{-30}$ (inert control group).

As shown in figure 1, animals treated with the test solution (black squares) metamorphosed more slowly than the control animals (white squares). The number of test animals that reached the four-legged stage at defined points in time was smaller in the group treated with homeopathically prepared thyroxin at most points in time. Differences were statistically significant ($p < 0.01$) at 3 points in time. Standard deviation was about + 2.5 to + 3.0 (a high value for SD is typical for hyperstimulated groups[6]. Animals treated with water $10^{-30}$ only (not hyperstimulized inert control group, black lines) were the slowest group.

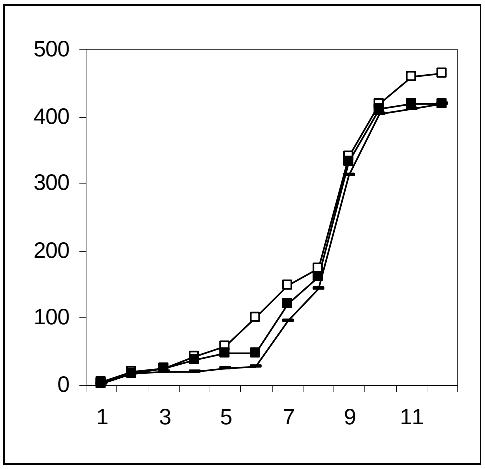

**Figure 2.** The effect of thyroxin $10^{-30}$, on lowland *Rana temporaria*, added from the spawn stage on. For further explanation, see legend to figure 1.

The data in figure 2 shows a difference of about 1-10%, i.e. frequencies of animals treated with homeopathically prepared thyroxin reached the values of the control animals with a delay of about 0.1 – 1 day (e.g. June 15th) or did not reach the values of the control animals at the end of the experiment respectively. P-values are given in table 1.

### DISCUSSION

In 1990, inspired by older literature on the influence of metal salts[11], we standardized an experimental model based on the influence of highly diluted thyroxin ($10^{-30}$) on inert Rana temporaria from highland biotopes. Results from a study in 1990[7] were confirmed by ourselves and others in 2000[6,9]: speed of metamorphosis was reduced by diluted thyroxin ($10^{-30}$) in all five laboratories involved.

In order to gain a physiological explication for these results, and further to optimize them, we added hyperstimulation with molecular thyroxin to our initial standard protocol.

**TABLE 1**
**For explanation see text**

| time (days) | hyp+WD30: hyp+TD30 | hyp+WD30: inert | hyp+TD30: inert |
|---|---|---|---|
| 1 | 0,48 | 0,48 | |
| 2 | 0,62 | 0,62 | |
| 3 | 0,77 | 0,37 | 0,54 |
| 4 | 0,57 | 0,00 | 0,02 |
| 5 | 0,26 | <0,001 | 0,01 |
| 6 | <0,001 | <0,001 | 0,01 |
| 7 | 0,05 | <0,001 | 0,07 |
| 8 | 0,46 | 0,06 | 0,24 |
| 9 | 0,64 | 0,06 | 0,17 |
| 10 | 0,56 | 0,25 | 0,57 |
| 11 | <0,001 | <0,001 | 0,61 |
| 12 | <0,001 | <0,001 | 0,93 |

Using such hyperstimulation, we were able to include animals from lowland biotopes into our study line. This was interesting with regard to the limited availability of highland amphibian. However, lowland animals, even when hyperstimulated, reacted to homeopathically prepared thyroxin only in 2/3 of experiments and, furthermore, only to a low dilution of thyroxin ($10^{-13}$). Thus, our hope to have found a robust experimental set-up involving *lowland* animals was deceived[6]. On the other hand, we learned a lot about the model, namely that if a difference between test group and control group is found in one laboratory at all, this difference is considerably greater when hyperstimulated animals than when inert animals are used[6]. This led to the possibility to explain our amphibian model as an intoxication-detoxification model analogous to other models used in homeopathy research[4].

From our studies we conclude that there appears to be a relationship between the effect of homeopathically prepared thyroxin and a naturally or artificially elevated thyroxin level in the animals during metamorphosis. It is reasonable to suppose that highland larves of Rana temporaria have become adapted to an environment which necessitates a comparatively high thyroxin level or high sensitivity to thyroxin[12,13]. This would be a plausible explanation for their consistent response in experiments with diluted thyroxin.

This assumption is backed by the observation that, in normal lowland animals, immersion in thyroxin $10^{-8}$ parts by weight leads to an increase of 4- legged animals of about 15% compared to immersion in plain basin water, whereas in highland animals, thyroxin $10^{-8}$ causes an increase of only around 5%[14].

Furthermore, the increase in environmental temperature causes an acceleration of metamorphosis when animals are brought from the highland biotopes to the laboratory. This acceleration is likely to be “cured” by homeopathically prepared thyroxin.

The present results permit the conclusion that administering homeopathically prepared thyroxin to amphibian larves during their thyroxin-controlled metamorphosis is in a certain sense analogous to the intoxication - detoxification concept used in other models of homeopathy research[4], although the intoxication dose and its effect on responsiveness do not seem to correlate in a linear way.

Apart from its inherent interest for physiology and medicine research, the amphibian model seems to be a helpful tool to investigate environmental influences on homeopathic drugs[14].

### ANNOTATION

The Interuniversity College would be pleased to collaborate with other institutions in order to prepare further independent repetitions of the experiment standardized in 1990 and repeated in 2000[7,9]. Such collaborations should include initial training at the laboratory of the College plus an independent experimental phase at the guest's own laboratory. Reprints of published papers can be downloaded at www.inter-uni.net/edition. The process of "trial and error", but also of "trial and success" in amphibian research has been described in the journalistic book "Homeopathy Research – An Expedition Report"[15].

## References

1. Roth, C. (1991) Literature review and critical analysis on the topic of in- and detoxication experiments in homoeopathy. *Berlin J Res Hom*. **1**, 111-117.
2. Herkovits, J., Perez-Coll, C., and Zeni, W. (1993) Reduced toxic effect of Cd on bufo arenarum embryos by means of very high diluted and stirred solutions of Cd. *Communicationes Biologicas* **7**, 70-73.
3. Weil, M.R. (1986) Changes in plasma thyroxin levels during and after spontaneous metamorphosis in a natural population of the green frog, Rana clamitans. Gen Comp Endocrinol. **62**, 8-12.
4. Göldner, C. (2005) Review und Evaluierung von neueren, mit homöopathischen Zubereitungen durchgeführten toxikologischen Experimenten. Dissertation, Graz University.
5. Guedes, J.R., Ferreira, C.M., and Guimaraes, H.M. et al. (2004) Homeopathically prepared solution of Rana catesbeiana thyroid glands modifies its rate of metamorphosis. *Homeopathy* **93**, 132-137.
6. Endler, P.C., Lüdtke, R., Heckmann, C., Zausner, C., Lassing, H., Scherer-Pongratz, W., Haidvogl, M., and Frass, M. (2003) Pretreatment with thyroxin ($10^{-8}$ parts by weight) enhances a "curative" effect of homeopathically prepared thyroxin ($10^{-13}$) on lowland frogs. *Res Compl Med.* **10**, 137-142.
7. Endler, P.C., Pongratz, W., Van Wijk, R., Kastberger, G., and Haidvogl, M. (1991) Effects of Highly Diluted Succussed Thyroxin on Metamorphosis of Highland Frogs. *Berlin J Res Hom.* **1**, 151-160.
8. Endler, P.C., Pongratz, W., Smith, C.W., and Schulte, J. (1995) Non-molecular information transfer from thyroxin to frogs With regard to homeopathic toxicology. *Vet Hum Tox.* **37**, 259-260.
9. Zausner, C., Lassnig, H., Endler, P.C., Scherer, W., Haidvogl, M., Frass, M., Kastberger G., and Lüdtke, R. (2002) Die Wirkung von "homöopathisch" zubereitetem Thyroxin auf die Metamorphose von Hochlandamphibien - Ergebnisse einer multizentrischen Kontrollstudie. *Perfusion* **17**, 268-276.
10. Welles, S.U., Suanjak-Traidl, E., Weber, S., Scherer-Pongratz, W., Frass, M., Endler, P.C., and Lothaller, H. (In Press) Does pretreatment with thyroxin (10e-8 M) enhance a "curative" effect of homeopathically prepared thyroxin (10e-30) on highland frogs? Results of a multi researcher study. *Res Compl Med*.
11. König, K. (1927) Über die Wirkung extreme verdünnter ("homöopathischer") Metallsalzlösungen auf Entwicklung und Wachstum von Kaulquappen. Zschft ges exp *Med.* **56**, 881-893.
12. Zausner-Lukitsch, C. (2001) Auswirkungen von homöopathisch zubereitetem Thyroxin auf die Metamorphosegeschwindigkeit von Rana temporaria. Thesis, Vienna University.
13. Endler, P.C. and Schulte, J. (1998) Ultra High Dilution. Physiology and Physics. Kluwer Academic Publishers, Dortrecht.
14. Welles, S.U., Weber, S., Suanjak-Traidl, E., Scherer-Pongratz, W., Frass, M., Endler, P.C., and Lothaller, H. (In Press) The effect of homeopathically prepared thyroxin ($10^{-30}$ parts by weight) on highland frogs is influenced by electromagnetic fields. *Homeopathy*.
15. Endler, P.C. (2003) Homeopathy - An Expedition Report. An old healing system gains plausibility. edition@inter-uni.net, Graz.
